# Skin pigmentation, sun exposure and vitamin D levels in children of the Avon Longitudinal Study of Parents and Children

**DOI:** 10.1186/1471-2458-14-597

**Published:** 2014-06-12

**Authors:** Carolina Bonilla, Andrew R Ness, Andrew K Wills, Debbie A Lawlor, Sarah J Lewis, George Davey Smith

**Affiliations:** 1 School of Social and Community Medicine, University of Bristol, Oakfield House, Oakfield Grove, Bristol BS8 2BN, UK; 2 School of Oral and Dental Sciences, University of Bristol, Bristol, UK; 3 MRC Integrative Epidemiology Unit (IEU) at the University of Bristol, Bristol, UK

**Keywords:** Pigmentation, Sun exposure, Vitamin D, ALSPAC, Genetic scores

## Abstract

**Background:**

It has been hypothesised that light skin pigmentation has arisen to ensure adequate levels of vitamin D as human populations moved out of Africa and into higher latitudes. Vitamin D, which is primarily obtained through exposure to sunlight (specifically ultraviolet radiation B (UVR-B)), has been inversely associated with several complex diseases. Greater sun exposure, on the other hand, is a well-known cause of skin cancer. The potential of UVR to be beneficial for some health outcomes but detrimental for others has prompted a public health debate on how to balance the positive and negative consequences of sun exposure. In this study we aimed to determine the validity of the evolutionary hypothesis linking lighter skin with higher vitamin D concentrations in a European population. Additionally, we aimed to examine the influence of pigmentation on personal behaviour towards sunlight exposure and the effects of this behaviour on vitamin D.

**Methods:**

We combined genetic variants strongly associated with skin colour, tanning or freckling to create genetic scores for each of these phenotypes. We examined the association of the scores with pigmentary traits, sun exposure and serum 25-hydroxyvitamin D (25(OH)D) levels among children of the Avon Longitudinal Study of Parents and Children (ALSPAC, N = 661 to 5649).

**Results:**

We found that fairer-skinned children, i.e. those with higher pigmentation score values, had higher levels of 25(OH)D (0.6 nmol/l; 95% CI 0.2, 1.0; per unit increase in skin colour score; N = 5649). These children also used more protection against the damaging effects of UVR.

**Conclusions:**

In this population taking protective measures against sunburn and skin cancer does not seem to remove the positive effect that having a less pigmented skin has on vitamin D production. Our findings require further replication as skin pigmentation showed only a small effect on circulating 25(OH)D.

## Background

Skin pigmentation is a complex phenotype that shows extensive variation between human populations. Evidence suggests that such variation was shaped by the action of natural selection at different latitudes, to prevent DNA damage by ultraviolet radiation (UVR) to the skin and to guarantee sufficient synthesis of vitamin D [1], although other hypotheses that explain its worldwide distribution have been proposed [2]. Among these are protection against light-induced folate deficiency, protection from cold injury, prevention of heat load, camouflage and resistance against infectious disease [2]. The vitamin D hypothesis posits that as human ancestors migrated out of the tropics and into areas with reduced sunlight there was a progressive depigmentation of the skin to allow for UVR-B (280–320 nm) induced vitamin D production [1].

Vitamin D synthesis is highly dependent on the concentration of melanin in the skin as melanin absorbs and scatters UVR-B, resulting in a less efficient conversion of 7-dehydrocholesterol to previtamin D_3_[3]. Therefore, dark-skinned individuals will experience slower vitamin D synthesis than light-skinned ones. This is especially important at higher latitudes where the incidence and duration of sunlight is reduced. However, although individuals with light skin pigmentation are more efficient at producing vitamin D, they will be affected by an elevated sunburn response, a lower tanning ability [4], and a greater susceptibility to skin cancers [5].

A number of studies have shown an association between skin lightness and 25-hydroxyvitamin D (25(OH)D) levels [6-10], most of them comparing individuals from different ethnicities.

Other factors that reduce the cutaneous photosynthesis of vitamin D are clothing and increasing age [3]. Sunscreens have been found to have a similar effect under strictly controlled conditions but recent evidence suggests that their normal use may not impair vitamin D production [11].

Because solar radiation constitutes the main source of vitamin D, but is also a risk factor for sunburn and skin carcinogenesis [12], achieving a balance between generating enough vitamin D while limiting the damage to the skin has become an important area of debate in public health (see for instance, [13]).

Several genes underlying human skin pigmentation and sun sensitivity have been identified through candidate and genome-wide association studies (GWAS) [14-18]. As a result, it is possible to combine polymorphisms from these genes to create genetic scores that are strongly associated with pigmentation traits, such as skin colour, tanning potential and freckling [19]. A score represents the additive effect of multiple genetic variants and is likely to account for a larger proportion of variance in the trait of interest than single genetic variants. The use of genetic scores instead of self- or interviewer-assessed pigmentation helps to avoid problems of measurement error and possible confounding.Using pigmentation genetic scores described earlier [19], we have examined associations of these scores with pigmentation phenotypes and sun exposure variables, and assessed their influence on serum 25(OH)D levels among UK children of the Avon Longitudinal Study of Parents and Children (ALSPAC). Our aim was to establish whether in this European population children with a lighter skin phenotype had higher circulating 25(OH)D as predicted by the vitamin D hypothesis for the evolution of skin pigmentation. Additionally, we aimed to examine the influence of pigmentation on personal behaviour towards sunlight exposure and whether this affects 25(OH)D levels.

## Methods

### Study population

ALSPAC is a population-based prospective cohort study investigating factors that affect the health and development of children and their parents. The study methods are described in detail elsewhere [20,21] (http://www.bristol.ac.uk/alspac/). In brief, pregnant women living around Bristol, South West England, who had an expected date of delivery between April 1991 and December 1992 were eligible, and of these 14,541 were enrolled in the study. Extensive data have been collected from the mothers and their offspring from pregnancy onwards by questionnaire, abstraction from medical notes, record linkage and by attendance at research clinics. Ethical approval for the study was obtained from the ALSPAC Law and Ethics Committee (IRB# 00003312) and the Local Research Ethics Committees (Bristol and Weston, Southmead, and Frenchay Health Authorities). Written informed consent was obtained from all participants in the study.

### Skin characteristics and sun exposure

A 10% randomly selected sample of parents whose babies were born within the last 6 months of the survey were invited to bring their children to a research clinic (Children in Focus, CIF) at 4, 8 and 12 months old and 6-monthly intervals thereafter up to 61 months of age, where a number of clinical, physiological and psychological assessments were completed. Skin colour was measured at 49 months of age with an EEL (Evans Electroselenium Ltd) spectrophotometer. Observers measured the skin reflectance on the inner surface of the arm, midway between scapula and elbow. The spectrophotometer was calibrated daily against a matt white ceramic tile and the interior of a black box to give 100% and 0% reflectances. The reflectance of a yellow melamine surface was then measured and recorded each day.

Detailed observations of freckles and moles were carried out at 49 months and repeated at 61 months. Before coming to the clinic at 49 months parents were given an outline drawing of a child of the same age and sex as their own, and asked to mark on it any freckles, moles or other marks on the skin. At the clinic the observers used this drawing as the focus when examining the child’s skin. They observed the child in good light, naked if possible. The number and location of all freckles and moles visible to the naked eye was noted on body maps on which face/scalp, neck, trunk and pelvis, and the upper, mid, and lower regions of each limb were shown, in both front and rear view (i.e. 30 areas of the body). Information on having any freckles at either 49 months, 61 months or both was combined in one exposure variable coded as 0 (no freckles at all) or 1 (at least one freckle at any age). Children with missing information on freckles at 49 months but with a valid response at 61 months were coded based on the latter. Children with no freckles at 49 months and missing information at 61 months were coded as missing. The number of moles (acquired melanocytic naevi) grouped in quartiles was used in the analysis. Mole count was additionally assessed during the clinic visit at age 15 years. A questionnaire was sent in advance asking for the presence of large and small moles, as measured by a plastic guide, to be indicated on arms and legs. Moles on one arm were validated on a sub-set of ~6% of children that attended the clinic. Total mole count was derived as the sum of the number of moles present on arms and legs.

For the whole of the ALSPAC cohort questions about the child’s previous exposure to the sun, the skin’s reaction, and protective measures taken, were included in the questionnaires sent to parents when the child was around 54, 65, 69, 77, 103 and 140 months old. A combined “sunburnt” variable was created using the information provided as a response to the question of whether the child had been badly sunburnt with blisters or pain for at least 2 days since the previous questionnaire instance. The variable was coded 0 (not badly sunburnt from birth until 140 months old) or 1 (at least one occurrence of bad sunburn up to 140 months of age). Children who did not report any instance of sunburn but who were missing information for one or more of the questionnaires were coded as missing.

Time spent outside in the sun annually either locally (more than 4 hours near the sea) or abroad at 5 and 9 years old was derived from questionnaires completed when the child was 69 and 103 months of age.

Additional file 1: Table S1 shows measured variables, time points at which they were collected and numbers of individuals available for each time point.

### Vitamin D levels

Serum total 25(OH)D (nmol/l) was assessed from samples obtained at mean age 9.9 years. If no samples were available from the assessment at age 9, which contained the largest number of children (N = 4364), samples from the 11 year assessment (N = 995) or the 7 year assessment (N = 1074) were used [22]. The age range of the children with a 25(OH)D measure was 85 to 163 months.

Following collection, samples were immediately spun, frozen and stored at -80°C, with no further freeze-thaw cycles to the time of 25(OH)D analysis. 25(OH)D_3_ and 25(OH)D_2_ were measured with high performance liquid chromatography tandem mass spectrometry using an internal standard in a laboratory meeting the performance target set by the Vitamin D External Quality Assessment Scheme (DEQAS) Advisory Panel for 25(OH)D assays. Inter-assay coefficients of variation for the assay were <10% across a working range of 2.5-624 nmol/l for both 25(OH)D_3_ and 25(OH)D_2_. Total 25(OH)D levels were calculated as the sum of 25(OH)D_3_ and 25(OH)D_2_[22]_._

### Genotyping

ALSPAC children were genotyped using the Illumina HumanHap550 quad chip (Illumina, Inc., San Diego, CA) by 23andMe subcontracting from the Wellcome Trust Sanger Institute, Cambridge, UK and the Laboratory Corporation of America, Burlington, NC, US. The resulting raw genome-wide data were subjected to standard quality control methods. Individuals were excluded on the basis of gender mismatches; minimal (<0.325) or excessive heterozygosity (>0.345); disproportionate levels of individual missingness (>3%), cryptic relatedness measured as proportion of identity by descent (IBD > 0.1) and insufficient sample replication (IBD < 0.8). The remaining individuals were assessed for evidence of population stratification by multidimensional scaling analysis and compared with Hapmap II (release 22) European descent (CEU), Han Chinese (CHB), Japanese (JPT) and Yoruba (YRI) reference populations; all individuals with non-European ancestry were removed. Single nucleotide polymorphisms (SNPs) with a minor allele frequency of < 1%, a call rate of < 95% or evidence for violations of Hardy-Weinberg equilibrium (p < 5×10^-7^) were removed. Genotypic data were subsequently imputed using Markov Chain Haplotyping software (MACH v.1.0.16) and phased haplotype data from CEU individuals (HapMap release 22, Phase II NCBI B36, dbSNP 126) based on a cleaned dataset of 8,365 individuals and 464,311 autosomal SNPs.

Genotypic dosages for all pigmentation-related SNPs of interest, which represent the expected number of one of the alleles and range from 0 to 2, were used in the association analysis. Genotypes were checked for deviation from Hardy-Weinberg equilibrium using the hwsnp function implemented in Stata (StataCorp LP, 2012, College Station, TX).

### Pigmentation genetic scores

In order to minimize bias due to residual confounding and measurement error of self- or interviewer-reported pigmentation and sun exposure variables, we computed three genetic scores from SNPs that have previously been associated with the pigmentation-related phenotypes examined in this study: skin colour (skin colour score = SCS), tanning ability (tanning score = TS) and freckling (freckling score = FS) [14]. It should be noted that ALSPAC samples were not part of any of the GWAS that discovered the pigmentation-associated SNPs and therefore the scores are unlikely to be overfitted in this independent sample. SNPs chosen had a minor allele frequency ≥ 0.05 in the CEU population, and the “risk” allele had been clearly established. Scores were calculated by summing the dosages of all appropriate SNPs in each individual, with no missing SNP data. “Risk” alleles were those associated with lighter skin color, burning rather than tanning, and a greater likelihood of having freckles. Dosages for each SNP range from 0 (when no risk alleles are present) to 2 (when the child carries two risk alleles).

Polymorphisms included in each score are shown in Table 1. These scores have been used before in a study of pigmentation characteristics, vitamin D and prostate cancer in British men [19].

**Table 1:** Skin colour, tanning and freckling genetic scores

**Skin colour score**					
**Gene**	**SNP**	**Chromosome**	**Chromosomal location**	**Major/minor alleles**	**Risk allele**^**a**^
*IRF4*	rs12203592	6p25.3	341321	C/T	T
*TYR*	rs1042602	11q14.3	88551344	C/A	A
*HERC2*	rs12913832	15q13.1	26039213	G/A	G
*MC1R*	rs1805007	16q24.3	88513618	C/T	T
*ASIP*	rs4911414	20q11.22	32193105	G/T	T
*ASIP*	rs1015362	20q11.22	32202273	G/A	G
**Tanning score**					
**Gene**	**SNP**	**Chromosome**	**Chromosomal location**	**Major/minor alleles**	**risk allele**^**b**^
*PPARGC1B*	rs32579	5q32	149191041	G/A	G
*IRF4*	rs12203592	6p25.3	341321	C/T	T
*IRF4/EXOC2*	rs12210050	6p25.3	420489	C/T	T
*TYR*	rs1393350	11q14.3	88650694	G/A	A
*CALCOCO1/HOXC13*	rs7969151	12q13.13	52445544	G/A	A
*PAPOLA/VRK1*	rs17094273	14q32.2	96173560	G/A	A
*HERC2*	rs12913832	15q13.1	26039213	G/A	G
*CPNE7/DPEP1*	rs154659	16q24.3	88194838	T/C	C
*MC1R*	rs1805007	16q24.3	88513618	C/T	T
*DBNDD1*	rs11648785	16q24.3	88612062	C/T	C
*ASIP*	rs4911414	20q11.22	32193105	G/T	T
*ASIP*	rs1015362	20q11.22	32202273	G/A	G
*PRDM15*	rs7279297	21q22.3	42100984	A/G	A
**Freckling score**					
**Gene**	**SNP**	**Chromosome**	**Chromosomal location**	**Major/minor alleles**	**Risk allele**^**c**^
*IRF4*	rs12203592	6p25.3	341321	C/T	T
*BNC2*	rs2153271	9p22.2	16854521	A/G	A
*TYR*	rs1042602	11q14.3	88551344	C/A	C
*TYR*	rs1393350	11q14.3	88650694	G/A	A
*MC1R*	rs1805007	16q24.3	88513618	C/T	T
*ASIP*	rs4911414	20q11.22	32193105	G/T	T
*ASIP*	rs1015362	20q11.22	32202273	G/A	G
*EIF6*	rs619865	20q11.22	33331111	G/A	A

^a^The risk allele is the allele associated with lighter skin pigmentation.

^b^The risk allele is the allele associated with higher propensity to burn.

^c^The risk allele is the allele associated with a greater likelihood of having freckles.

### Population stratification

The top 10 principal components (PCs) that reflect the population’s genetic structure were estimated according to Price et al. [23] from genome-wide SNPs genotyped, imputed and cleaned in ALSPAC children as described above. All 10 PCs were included as covariates in the regression models to account for confounding by population stratification.

### Potential confounders

We examined whether the genetic scores were associated with confounders. Children’s date of birth and sex were obtained from birth records. Information on maternal education was collected using questionnaires completed by the mothers at approximately 18 and 32 weeks of pregnancy.

### Statistical analysis

The main source of 25(OH)D_3_ (and hence total 25(OH)D) is synthesis in the skin in response to UVR-B exposure, which means there is a strong sinusoidal pattern of 25(OH)D with date of blood collection. We modelled this relationship using a cosine regression model and extracted the residuals, using these as our main 25(OH)D measurement. These residuals remove the seasonal influence on 25(OH)D and are a better marker of habitual 25(OH)D concentration for each individual, they also remove the noise in the 25(OH)D measure associated with the quasi-random sampling dates [24].

The association of genetic scores with pigmentation and sun exposure variables, and serum 25(OH)D levels, was assessed using one way ANOVA, t-tests, linear and logistic regression with adjustment for age, sex and population stratification. Interaction between genetic scores and sex on reflectance and freckling phenotypes was examined using a likelihood ratio test. The unique contribution of each genetic score to the variability of skin reflectance, freckling and serum 25(OH)D was calculated as the square of the semipartial correlation coefficients obtained from a model that additionally includes sex, age at clinic visit and population stratification principal components.

## Results

### Pigmentation genetic scores

One SNP, rs7279297, was marginally out of Hardy-Weinberg equilibrium (p = 0.05).

All scores were normally distributed. The skin colour score ranged from 1 to 10, the tanning score from 4 to 19, and the freckling score from 1 to 13, a higher score indicating lighter skin colour, skin that burns instead of tanning, and a greater chance of having freckles. The correlation of scores after adjustment for population stratification was: TS vs SCS, R^2^ = 0.19; TS vs FS R^2^ = 0.22; SCS vs FS R^2^ = 0.01; p < 0.001 for all comparisons. This correlation partly reflects the fact that some SNPs are present in more than one score. The correlation of scores with principal components representative of population stratification is shown in Additional file 2: Table S2.

### Potential confounders

None of the genetic scores were associated with sex (Additional file 1: Table S3) or maternal education (Additional file 1: Table S4). The skin colour score was weakly associated with age of the child at completion of the 54 month questionnaire (mean difference per unit increase in score: 0.02 months; 95% CI 0.001, 0.05; p = 0.04), the other genetic scores were not associated with age.

Sex was associated with skin reflectance, skin colour change after spending time in the sun, having been badly burnt, mole count at ~5 and 15 years of age and wearing protective clothing and sun block when outside (Additional file 1: Table S3). Some of these findings suggested that girls were slightly darker than boys (e.g. showing lower skin reflectance), although other results appeared inconsistent with this conclusion (e.g. a lower number of girls reported tanning easily or always tanning). Time spent in the sun at ~5 and ~9 years old, whether in Britain or overseas, did not differ between girls and boys (Additional file 1: Table S3). Sex was associated with serum 25(OH)D, with girls having on average lower levels than boys (mean difference: -3.41 nmol/l; 95% CI -6.34, -0.48; p = 0.02, adjusted for age at blood draw). There was a similar difference between sexes after additional adjustment for skin reflectance and sun block use (mean difference: -3.53 nmol/l; 95% CI -6.52, -0.55; p = 0.02).

Skin colour change after being in and out of the sun for a few days, experiencing a bad burn, wearing protective clothing and sun block, and time spent outside locally or abroad were associated with maternal education, with a tendency for children of better educated mothers to burn easily (although having a bad burn less often), frequently use sun protection and spend more time in the sun. In addition, circulating 25(OH)D was marginally lower for children of mothers with a high educational achievement (Additional file 1: Table S4).

### Pigmentation genetic scores and sun exposure variables

Pigmentation scores were robustly associated with skin colour (as measured by skin reflectance, Table 2), tanning ability, skin reaction, freckling and mole count (Table 3), in the directions expected. Moreover, scores were associated with patterns of behaviour that may reflect having a certain skin complexion, such as wearing protective clothing or applying sun block (Additional file 1: Table S5). The effect of the freckling score was weaker with respect to the behavioural variables compared to the effects of the skin colour and tanning scores. Children with higher scores (i.e. lighter skin colour, who tend to burn and have freckles) were more likely to always be covered or use sun block when out in the sun than children with lower scores. Adjustment for population stratification did not alter the results appreciably (data available on request).

**Table 2:** Pigmentation scores and skin reflectance

**Pigmentation scores**	**Effect**^**a**^**(unadjusted)**	**95% CI**	**p-value**	**R**^**2**^	**R**^**2**^**(%)**	**F**	**Effect**^**a**^**(adjusted**^**b**^**)**	**95% CI**	**p-value**
Skin colour score	0.41	0.25, 0.57	8.1×10^-7^	0.035	3.46	24.8	0.41	0.25, 0.57	7.6×10^-7^
Tanning score	0.19	0.10, 0.28	5.6×10^-5^	0.023	2.32	16.4	0.18	0.09, 0.27	9.3×10^-5^
Freckling score	0.25	0.13, 0.36	1.9×10^-5^	0.026	2.62	18.6	0.22	0.11, 0.34	1.0×10^-4^

^a^Mean difference in skin reflectance per unit increase in pigmentation score.

^b^Adjusted for sex, age at clinic visit and principal components.

N = 693.

**Table 3:** Distribution of reported or observed pigmentation characteristics and 25(OH)D levels by pigmentation genetic scores

	**Mean ± SD of pigmentation score**				**Mean ± SD**	
	**Skin colour score**	**Tanning score**	**Freckling score**	**N**^**a**^	**25(OH)D (nmol/l)**	**N**^**b**^
**Skin colour change**						
Always burns never tans	6.29 ± 1.25	12.16 ± 1.97	7.16 ± 1.54	47	62.8 ± 14.6	45
Burns easily rarely tans	5.69 ± 1.27	11.76 ± 2.19	6.82 ± 1.81	547	63.4 ± 16.7	518
Doesn’t change	5.41 ± 1.18	11.19 ± 2.02	6.41 ± 1.66	235	64.9 ± 18.4	225
Tans easily rarely burns	4.89 ± 1.17	10.35 ± 2.00	5.85 ± 1.61	2209	63.2 ± 19.9	2098
Always tans never burns	4.57 ± 1.11	9.92 ± 1.95	5.56 ± 1.54	924	63.6 ± 20.1	875
p-value	<0.0001	<0.0001	<0.0001	3962	0.81/0.53^c^	3761
**Child badly burnt**						
No	5.00 ± 1.21	10.54 ± 2.08	5.96 ± 1.68	3501	62.4 ± 18.7	3465
Yes	5.40 ± 1.29	11.27 ± 2.17	6.42 ± 1.70	613	63.7 ± 19.3	594
p-value	1.5×10^-13^	2.2×10^-15^	6.4×10^-10^	4114	0.13/0.17^c^	4059
**Any freckles**						
No	4.70 ± 1.14	10.06 ± 1.96	5.52 ± 1.57	432	60.8 ± 19.1	391
Yes	5.45 ± 1.23	11.41 ± 2.18	6.79 ± 1.80	297	63.7 ± 19.2	270
p-value	2.0×10^-16^	2.1×10^-17^	2.3×10^-22^	729	0.05/0.04^c^	661
**Mole count**						
**49 months**						
Q1 (0–6)	4.84 ± 1.17	10.27 ± 1.90	5.80 ± 1.60	227	61.3 ± 16.6	193
Q2 (7–9)	4.94 ± 1.18	10.30 ± 2.04	5.66 ± 1.64	144	60.2 ± 15.4	123
Q3 (10–14)	5.03 ± 1.25	10.75 ± 2.22	6.08 ± 1.95	186	61.5 ± 25.2	166
Q4 (15–57)	5.37 ± 1.22	11.24 ± 2.27	6.49 ± 1.80	176	62.8 ± 16.7	169
p-value	0.0002	1.89×10^-6^	3.16×10^-5^	733	0.71/0.74^c^	651
**61 months**						
Q1 (0–10)	4.83 ± 1.22	10.07 ± 2.06	5.55 ± 1.75	191	60.4 ± 17.6	164
Q2 (11–16)	4.84 ± 1.24	10.43 ± 2.11	5.90 ± 1.67	174	60.5 ± 21.7	162
Q3 (17–25)	5.05 ± 1.17	10.68 ± 2.16	6.23 ± 1.82	180	61.4 ± 15.1	163
Q4 (26–86)	5.30 ± 1.25	11.21 ± 2.10	6.43 ± 1.73	166	65.2 ± 21.5	160
p-value	0.001	3.50×10^-7^	4.26×10^-7^	711	0.08/0.09^c^	649
**186 months**						
Q1 (0–2)	5.05 ± 1.22	10.56 ± 2.15	6.05 ± 1.76	688	60.6 ± 17.0	704
Q2 (3–9)	4.92 ± 1.20	10.50 ± 2.14	6.03 ± 1.72	691	61.4 ± 19.1	717
Q3 (10–22)	5.00 ± 1.21	10.60 ± 2.08	6.01 ± 1.67	710	62.3 ± 18.5	729
Q4 (23–153)	5.21 ± 1.18	10.83 ± 2.04	6.03 ± 1.65	665	63.5 ± 18.5	656
p-value	0.0001	0.02	0.99	2754	0.02/0.01^c^	2806

^a^N = number of individuals included in the analysis of pigmentation scores.

^b^N = number of individuals included in the analysis of 25(OH)D levels.

^c^Adjusted for sex and age at blood draw.

Except for the tanning score and spending time in the sun when abroad at 9 years of age (mean difference in tanning score for children who spent time in the sun -0.35; 95% CI -0.61, -0.08; p = 0.01), there were no associations between the pigmentation scores and whether children spent time in the sun (data available on request).

There was no clear evidence of an interaction between the skin colour score and sex on skin reflectance (p for interaction = 0.12), but we found some evidence of an interaction between the freckling score and sex on having any freckles (p for interaction = 0.02). The association of the genetic score with the pigmentation phenotype was stronger in girls for all scores and traits, with the exception of the effect of the freckling score on reflectance which was considerably larger in boys (Additional file 1: Tables S6 and S7). The unique contribution of each genetic score to total variance in skin reflectance and having any freckles was between 1.5 and 4 times greater in girls than in boys (Additional file 1: Tables S6 and S7).

### Sun exposure variables and vitamin D levels

Children who were reported to never use sun block when out in the sun exhibited lower 25(OH)D levels compared to children who always did so (Additional file 1: Table S5). Children with freckles, and with a greater number of moles at ages 5 and 15 (Table 3) had higher serum 25(OH)D as did those who spent time in the sun abroad (Additional file 1: Table S8). There was no association of skin colour change after being in the sun, having experienced a bad sunburn, mole count at age 4, wearing clothing when outside, and sun block factor used, with 25(OH)D concentration (Table 3 and Additional file 1: Table S5). Likewise, skin reflectance was not observationally associated with 25(OH)D, (mean difference per unit increase in reflectance: 0.12 nmol/l; 95% CI -0.45, 0.68; p = 0.68; adjusted for sex and age at blood draw).

### Pigmentation genetic scores and vitamin D levels

The pigmentation genetic scores were associated with serum 25(OH)D, i.e. the lighter the skin (as indicated by the genetic score) the higher the circulating 25(OH)D levels (Table 4). Additional adjustment of each score for the other two showed an association of the skin colour score only (mean difference per unit increase in skin colour score: 0.5 nmol/l; 95% CI 0.1, 1.0; p = 0.03). Scores explained a small proportion of the variability in 25(OH)D levels (R^2^ < 0.002).

**Table 4:** Pigmentation scores and 25(OH)D levels

**Pigmentation scores**	**25(OH)D (nmol/l)**^**a**^	**95% CI**	**p-value**	**R**^**2b**^
Skin colour score	0.6	0.2, 1.0	0.003	0.0015
Tanning score	0.3	0.1, 0.6	0.003	0.0014
Freckling score	0.4	0.1, 0.7	0.01	0.0012

^a^Mean difference in 25(OH)D levels per unit increase in pigmentation score, adjusted for sex, age at blood draw and principal components.

^b^Semipartial correlation coefficient squared, indicates the unique contribution of the genetic score to trait variability.

N = 5649.

## Discussion

Our results show that in this European population fairer skin was linked to higher concentrations of 25(OH)D, in agreement with the vitamin D hypothesis for the evolution of skin pigmentation. We used genetic scores that are robustly associated with pigmentation traits and sun exposure measurements in order to reduce bias due to self-report or clinical assessment. Whilst age, sex and maternal education were all associated with one or more reported or assessed pigmentation phenotypes and also associated with 25(OH)D, they were mostly unassociated with our genetic scores, supporting the use of genetic scores as unbiased and unconfounded proxies for pigmentary features. In cohorts with genotypic data available but no sun exposure measurements or low quality sun exposure information we could use genetic scores to assess the relationship between sun exposure and an outcome of interest.

In our study population higher scores corresponded to higher skin reflectance (i.e. lighter skin colour), a greater likelihood of having freckles, a higher mole count, a skin that always burns and never tans, having experienced at least one bad sunburn, always covering the skin with clothing or sun block when out in the sun, and using sun lotion with a high sun protection factor (SPF). The skin colour score, for instance, explained ~3.5% of the variability in skin reflectance among ALSPAC children.

The association of the genetic scores with sun exposure phenotypes confirms previous results obtained in British men who participated in the Prostate Testing for cancer and Treatment (ProtecT) study, where the same pigmentation scores were used [19].

The pigmentation scores, in particular the skin colour score, were associated with 25(OH)D levels, with higher scores predicting higher serum 25(OH)D, despite being associated with behaviours that would reduce the impact of UVR exposure on vitamin D synthesis. However, the magnitude of the effect was small. Although darker children (i.e. those with lower pigmentation scores) had lower 25(OH)D levels on average, only 0.5% of children were vitamin D deficient in this population (serum 25(OH)D < 25 nmol/l [25]). These results do not concur with our findings in the ProtecT study which showed that British men with lighter skins –as suggested by their genetic scores- had lower circulating 25(OH)D [19], or with a study of British women in which lighter skin pigmentation (assessed by nurses using the Fitzpatrick scale) was also associated with lower levels of 25(OH)D [26]. These individuals possibly used more abundant sun protection or restricted their exposure to the sun. Children not wearing enough or effective sun protection and/or spending more time outside than adults may explain the reverse association.

There were clear differences by sex in reported sun exposure patterns as well as in pigmentation traits that suggested that girls were to some extent darker than boys. Yet, although apparently more protected from sunburn, girls reported more consistently applying sun block (or having sun block applied on them) when outdoors than boys. Time spent outside in the sun was similar for boys and girls. Girls have been found to have darker skin than boys in pre-adolescence, with boys becoming darker than girls around puberty [2,27]. In our study there were more girls than boys with a low mole count at 4–5 years of age but this pattern appeared to have reversed by 15 years. In adults, across a range of countries and ethnicities, men tend to be more pigmented than women [2,28], however, lighter-skinned men were identified in a study carried out in four European countries[18], in a sample of European Americans [29], and amongst populations in the Netherlands, Belgium and the Basque Country [20,21].

On the other hand, mean pigmentation genetic scores did not vary between sexes, suggesting that the observed gender differences in pigmentation phenotypes may be explained by environmental, hormonal or other genetic causes. For instance, it is well-known that cutaneous pigmentation is modulated by growth factors, cytokines and endocrine hormones, which stimulate melanocyte proliferation and⁄or melanogenesis [30,31].

The association of the pigmentation scores with skin reflectance and having freckles was for the most part stronger in girls (with a higher reflectance and likelihood of freckling as the pigmentation score increases towards the lighter phenotype), accounting for about twice the phenotypic variance explained by boys’ scores. A possible reason for this finding may be sexual selection by men for lighter-skinned women during human evolution [27,32]. In fact, a few of the SNPs included in the pigmentation scores are located in genes that have been targets of selection (i.e. *MC1R*, *HERC2*, *TYR*, *ASIP*) [33-35]. However, because we concurrently found lower reflectance values in girls, other factors could be influencing skin colour in the opposite direction in the pre-pubertal stages. A recent study on mate preferences among UK twins revealed a patent sexually dimorphic preference for skin colour but, unexpectedly, women preferred fair-skinned men whilst men showed a preference for women with olive skin. Although a familial environmental effect was uncovered, no genetic effects on mate preference for skin colour were evident in this study [36]. These sex differences deserve to be examined more carefully in the future as they may have an impact on the onset and progression of melanoma [37]. Alternatively, given our small sample size and the lack of strong evidence for statistical heterogeneity between girls and boys, the observed sex disparities may simply be due to chance.

Differences in sun exposure-related variables were also detected with respect to maternal educational achievement, such that children of better educated mothers were more likely to burn rather than tan, wear protective clothing and use sun block, as well as were more often exposed to the sun in Britain or abroad. These children were also found to have slightly lower 25(OH)D levels. These findings possibly reflect reporting bias since educated mothers are usually more aware of the risks and benefits posed by the sun, and can also afford sun protection and summer holidays. Similar results with respect to educational level were reported in an observational study that assessed the relationship between sun exposure habits, sun protection behaviour and readiness to increase sun protection in an adult primary health care population in Sweden. Subjects of lower educational attainment reported being sunburnt less frequently, used less sun protection and revealed a lower propensity to increase sunscreen use [38].

### Limitations

Some of the pigmentation and sun exposure variables were measured only once in children between birth and age 17 (latest data release on ALSPAC children at the time of analysis) and therefore, it was not possible to assess their variation with age or to examine the contribution of pigmentation genes to these exposures at different ages. Other variables were measured at time points that were close together so were not very informative about age-related variation either. Time spent outside was only analysed based on having holidays near the sea or abroad and not with basis on the children’s regular outdoor experience. Given that in the 90s, when ALSPAC children were growing up, there was less awareness of UVR exposure risks and the industry of sun protection was less developed -hence the low sun block SPFs available- parents’ behaviour towards their offspring sunlight exposure may have been different from that of present-day parents of young children [39]. Therefore, findings from this study may not be fully generalisable to the present day.

Because some SNPs were associated with more than one pigmentation trait, they were included in more than one score and therefore, scores were correlated. Scores explained some of the variation in the non-specific traits, but the strongest association was seen with the specific phenotypes. Since we were mostly interested in assessing the influence of a lighter skin phenotype overall on sun exposure measurements and 25(OH)D levels, and not to unequivocally establish the effects of each specific pigmentation trait, the correlation between scores does not bias our conclusions. However, we acknowledge that to understand the role of specific pigmentation traits (i.e. skin colour, tanning and freckling) on the efficiency of vitamin D synthesis other approaches would be needed [40,41].

We had limited statistical power to examine the association of some of the sun exposure variables with the genetic scores and with 25(OH)D levels, especially with respect to the interaction analyses, consequently the evaluation of these associations in larger studies is essential.

## Conclusions

We have found that children with a lighter skin phenotype (light skin colour, burning, and freckles, as suggested by their genetic scores) had higher circulating 25(OH)D than their peers, in spite of the fact that they were more likely to wear sun protective clothing or sun block. These findings concur with the vitamin D hypothesis for the evolution of skin pigmentation, and indicate that the benefits of having a less pigmented skin at a northern latitude do not seem to be eradicated by the use of sunscreen or clothing in UK children. However, we did not have enough information to establish whether the sun avoidance behaviour of ALSPAC children was indeed protective from UVR damage. If greater protection is deemed necessary in children to prevent sunburn and skin cancer, its effects on 25(OH)D levels may become more relevant. In contrast, as reported in an earlier study, UK white men with lighter skin showed lower concentrations of 25(OH)D [19]. Our results suggest that only a small proportion of the variation in 25(OH)D is explained by the pigmentation genetic scores and thus require replication in other studies of children before we can conclude that they are robust.

Lastly, we have also shown that personal behaviour towards the sun is affected by an individual’s pigmentary features, for instance, children of lighter complexion were more likely to always cover their skin with clothing, always wear sun block, and to apply sun block with an SPF 25 or higher.

## Competing interests

The authors declare they have no competing interests.

## Authors’ contributions

CB, GDS and DAL conceived the study. CB performed the analysis and drafted the manuscript. ARN participated in mole questionnaire design and data collection. AKW participated in vitamin D data analysis. SJL critically reviewed earlier versions of the manuscript. All authors reviewed and approved the final version.

## Pre-publication history

The pre-publication history for this paper can be accessed here:

http://www.biomedcentral.com/1471-2458/14/597/prepub

## Supplementary Material

Additional file 1: Table S1 Measures of sun exposure and skin reaction obtained at different time points during childhood in ALSPAC children. Figures given correspond to number of individuals with the specific measure. **Table S3.** Sun exposure variables and pigmentation genetic scores in ALSPAC boys and girls. **Table S4.** Sun exposure variables and pigmentation genetic scores according to maternal education level. **Table S5.** Pigmentation genetic scores and 25(OH)D levels according to sun avoidance behaviour in ALSPAC children. **Table S6.** Association of pigmentation genetic scores with skin reflectance stratified by sex. **Table S7.** Association of pigmentation genetic scores with having freckles, stratified by sex. **Table S8.** Time spent in the sun locally or abroad and 25(OH)D levels. Click here for file

Additional file 2: Table S2 Pigmentation genetic scores and population structure in ALSPAC children. Click here for file
