# Supplementary material for: Skin pigmentation, sun exposure and vitamin D levels in children of the Avon Longitudinal Study of Parents and Children
Source: BMC Public Health. 2014 Jun 12;14:597. doi: 10.1186/1471-2458-14-597 (PMC4067096; doi:10.1186/1471-2458-14-597)
Supplement: Additional file 1: Table S1 — Measures of sun exposure and skin reaction obtained at different time points during childhood in ALSPAC children. Figures given correspond to number of individuals with the specific measure. Table S3. Sun exposure variables and pigmentation genetic scores in ALSPAC boys and girls. Table S4. Sun exposure variables and pigmentation genetic scores according to maternal education level. Table S5. Pigmentation genetic scores and 25(OH)D levels according to sun avoidance behaviour in ALSPAC children. Table S6. Association of pigmentation genetic scores with skin reflectance stratified by sex. Table S7. Association of pigmentation genetic scores with having freckles, stratified by sex. Table S8. Time spent in the sun locally or abroad and 25(OH)D levels. [file 1471-2458-14-597-S1.docx]

**Supplementary Table 1.** Measures of sun exposure and skin reaction obtained at different time points during childhood in ALSPAC children. Figures given correspond to number of individuals with the specific measure.

| **measure\months** | **49** | **54** | **61** | **65** | **69** | **77** | **90** | **103** | **118** | **140** | **186** |
| --- | --- | --- | --- | --- | --- | --- | --- | --- | --- | --- | --- |
| skin reflectance | 878 |  |  |  |  |  |  |  |  |  |  |
| freckles | 933 |  | 890 |  |  |  |  |  |  |  |  |
| skin colour change |  |  |  |  | 5493 |  |  |  |  |  |  |
| bad sunburn |  | 8712 |  | 8156 | 7800 | 7773 |  | 7184 |  | 6524 |  |
| covering skin |  |  |  |  | 7701 |  |  |  |  |  |  |
| wearing sun block |  |  |  |  | 7758 |  |  |  |  |  |  |
| sun block factor |  |  |  |  | 7500 |  |  |  |  |  |  |
| time in sun locally |  |  |  |  | 6398 |  |  | 1588 |  |  |  |
| time in sun overseas |  |  |  |  | 6402 |  |  | 1790 |  |  |  |
| mole count | 933 |  | 890 |  |  |  |  |  |  |  | 3257 |
| 25(OH)D (nmol/l) |  |  |  |  |  |  | 1074 |  | 4364 | 995 |  |

**Supplementary Table 3.** Sun exposure variables and pigmentation genetic scores in ALSPAC boys and girls.

|  | **males (%)** | **females (%)** | **p-value** |
| --- | --- | --- | --- |
| **skin colour change** |  |  | 0.001 |
| always burns never tans | 1.3 | 1.4 |  |
| burns easily rarely tans | 12.4 | 14.6 |  |
| doesn’t change | 4.7 | 6.7 |  |
| tans easily rarely burns | 57.0 | 54.5 |  |
| always tans never burns | 24.6 | 22.8 |  |
| N | 2921 | 2572 |  |
|  |  |  |  |
| **child badly burnt** |  |  | <0.001 |
| no | 82.5 | 86.3 |  |
| yes | 17.6 | 13.7 |  |
| N | 2724 | 2584 |  |
|  |  |  |  |
| **any freckles** |  |  | 0.97 |
| no | 58.4 | 58.2 |  |
| yes | 41.6 | 41.8 |  |
| N | 502 | 419 |  |
|  |  |  |  |
| **mole count in quartiles** |  |  |  |
| **49 months** |  |  | 0.42 |
| Q1 (0 -6) | 30.2 | 32.1 |  |
| Q2 (7 - 9) | 18.7 | 21.9 |  |
| Q3 (10 - 14) | 24.8 | 23.1 |  |
| Q4 (15 - 57) | 26.3 | 22.9 |  |
| N | 513 | 420 |  |
|  |  |  |  |
| **61 months** |  |  | 0.02 |
| Q1 (0 -10) | 23.7 | 28.5 |  |
| Q2 (11 - 16) | 22.3 | 27.8 |  |
| Q3 (17 - 25) | 26.9 | 23.7 |  |
| Q4 (26 - 86) | 27.1 | 20.0 |  |
| N | 480 | 410 |  |
|  |  |  |  |
| **186 months** |  |  | <0.001 |
| Q1 (0 - 2) | 26.8 | 23.6 |  |
| Q2 (3 – 9) | 26.3 | 23.8 |  |
| Q3 (10 – 22) | 26.6 | 25.2 |  |
| Q4 (23 – 153) | 20.3 | 27.4 |  |
| N | 1565 | 1692 |  |
|  |  |  |  |
| **covering skin** |  |  | <0.001 |
| always | 24.2 | 21.5 |  |
| usually | 47.7 | 44.0 |  |
| sometimes | 27.2 | 32.5 |  |
| never | 0.9 | 2.0 |  |
| N | 3970 | 3731 |  |
|  |  |  |  |
| **wearing sun block** |  |  | 0.01 |
| always | 58.7 | 62.1 |  |
| usually | 31.5 | 29.7 |  |
| sometimes | 8.9 | 7.5 |  |
| never | 0.9 | 0.7 |  |
| N | 3994 | 3764 |  |
|  |  |  |  |
| **sun block factor** |  |  | 0.37 |
| 1-3 | 0.2 | 0.1 |  |
| 4-7 | 1.0 | 1.5 |  |
| 8-14 | 7.2 | 7.0 |  |
| 15-20 | 35.4 | 35.4 |  |
| 21-25 | 18.5 | 17.9 |  |
| >25 | 37.7 | 38.1 |  |
| N | 3758 | 3647 |  |
|  |  |  |  |
| **time in sun near sea at 5 years old** |  |  | 0.99 |
| no | 24.0 | 23.9 |  |
| yes | 76.0 | 76.1 |  |
| N | 3265 | 3133 |  |
|  |  |  |  |
| **time in sun abroad at 5 years old** |  |  | 0.65 |
| no | 68.9 | 68.3 |  |
| yes | 31.1 | 31.7 |  |
| N | 3279 | 3123 |  |
|  |  |  |  |
| **time in sun near sea at 9 years old** |  |  | 0.57 |
| no | 37.4 | 38.8 |  |
| yes | 62.6 | 61.2 |  |
| N | 827 | 761 |  |
|  |  |  |  |
| **time in sun abroad at 9 years old** |  |  | 0.81 |
| no | 76.0 | 75.6 |  |
| yes | 24.0 | 24.4 |  |
| N | 935 | 855 |  |
|  |  |  |  |
| **skin reflectance** |  |  | <0.0001 |
| mean ± SD | 75.6 ± 2.7 | 74.6 ± 2.7 |  |
| N | 496 | 382 |  |
|  |  |  |  |
| **skin colour score** |  |  | 1.00 |
| mean ± SD | 5.05 ± 1.21 | 5.05 ± 1.23 |  |
| N | 3921 | 3702 |  |
|  |  |  |  |
| **tanning score** |  |  | 0.85 |
| mean ± SD | 10.64 ± 2.11 | 10.63 ± 2.13 |  |
| N | 3921 | 3702 |  |
|  |  |  |  |
| **freckling score** |  |  | 0.40 |
| mean ± SD | 6.05 ± 1.73 | 6.02 ± 1.68 |  |
| N | 3921 | 3702 |  |
|  |  |  |  |
| **25(OH)D (nmol/l)** |  |  | <0.0001 |
| mean ± SD | 65.0 ± 20.8 | 61.2 ± 18.1 |  |
| N | 3262 | 3156 |  |

**Supplementary Table 4.** Sun exposure variables and pigmentation genetic scores according to maternal education level.

|  | **< O level (%)** | **O level (%)** | **> O level (%)** | **p-value** |
| --- | --- | --- | --- | --- |
| **skin colour change** |  |  |  | <0.001 |
| always burns never tans | 1.9 | 1.1 | 1.2 |  |
| burns easily rarely tans | 11.3 | 13.4 | 15.0 |  |
| doesn’t change | 5.2 | 5.2 | 6.5 |  |
| tans easily rarely burns | 53.3 | 56.8 | 56.6 |  |
| always tans never burns | 28.3 | 23.5 | 20.7 |  |
| N | 1314 | 1980 | 2075 |  |
|  |  |  |  |  |
| **child badly burnt** |  |  |  | <0.001 |
| no | 80.4 | 84.7 | 86.1 |  |
| yes | 19.6 | 15.3 | 13.9 |  |
| N | 1044 | 1844 | 2351 |  |
|  |  |  |  |  |
| **any freckles** |  |  |  | 0.78 |
| no | 60.3 | 58.7 | 57.2 |  |
| yes | 39.7 | 41.3 | 42.8 |  |
| N | 189 | 322 | 393 |  |
|  |  |  |  |  |
| **mole count in quartiles** |  |  |  |  |
| **49 months** |  |  |  | 0.83 |
| Q1 (0 -6) | 31.6 | 28.7 | 32.1 |  |
| Q2 (7 - 9) | 19.4 | 20.7 | 20.0 |  |
| Q3 (10 - 14) | 21.9 | 24.8 | 25.4 |  |
| Q4 (15 - 57) | 27.1 | 25.8 | 22.5 |  |
|  |  |  |  |  |
| **61 months** |  |  |  | 0.59 |
| Q1 (0 -10) | 25.8 | 27.5 | 25.1 |  |
| Q2 (11 - 16) | 24.2 | 21.5 | 28.5 |  |
| Q3 (17 - 25) | 25.8 | 26.6 | 24.0 |  |
| Q4 (26 - 86) | 24.2 | 24.4 | 22.4 |  |
|  |  |  |  |  |
| **186 months** |  |  |  | 0.17 |
| Q1 (0 - 2) | 27.4 | 25.8 | 23.7 |  |
| Q2 (3 – 9) | 25.0 | 23.1 | 26.7 |  |
| Q3 (10 – 22) | 22.7 | 27.2 | 25.9 |  |
| Q4 (23 – 153) | 24.9 | 23.9 | 23.7 |  |
|  |  |  |  |  |
| **covering skin** |  |  |  | <0.001 |
| always | 29.5 | 23.3 | 18.7 |  |
| usually | 40.5 | 45.5 | 49.4 |  |
| sometimes | 28.0 | 29.9 | 30.7 |  |
| never | 2.0 | 1.3 | 1.2 |  |
| N | 1781 | 2702 | 3052 |  |
|  |  |  |  |  |
| **wearing sun block** |  |  |  | <0.001 |
| always | 66.2 | 62.3 | 54.8 |  |
| usually | 23.0 | 29.9 | 36.1 |  |
| sometimes | 9.5 | 7.1 | 8.5 |  |
| never | 1.3 | 0.7 | 0.6 |  |
| N | 1796 | 2722 | 3073 |  |
|  |  |  |  |  |
| **sun block factor** |  |  |  | 0.09 |
| 1-3 | 0.4 | 0.1 | 0.1 |  |
| 4-7 | 1.6 | 1.1 | 1.2 |  |
| 8-14 | 6.2 | 7.5 | 7.1 |  |
| 15-20 | 36.9 | 34.8 | 35.3 |  |
| 21-25 | 16.9 | 18.3 | 18.9 |  |
| >25 | 38.0 | 38.2 | 37.4 |  |
| N | 1668 | 2660 | 3012 |  |
|  |  |  |  |  |
| **time in sun near sea at 5 years old** |  |  |  | <0.001 |
| no | 30.2 | 23.9 | 20.5 |  |
| yes | 69.8 | 76.1 | 79.5 |  |
| N | 1422 | 2248 | 2592 |  |
|  |  |  |  |  |
| **time in sun abroad at 5 years old** |  |  |  | <0.001 |
| no | 75.0 | 71.0 | 62.6 |  |
| yes | 25.0 | 29.0 | 37.4 |  |
| N | 1469 | 2266 | 2537 |  |
|  |  |  |  |  |
| **time in sun near sea at 9 years old** |  |  |  | <0.001 |
| no | 45.6 | 37.4 | 33.2 |  |
| yes | 54.4 | 62.6 | 66.8 |  |
| N | 388 | 577 | 579 |  |
|  |  |  |  |  |
| **time in sun abroad at 9 years old** |  |  |  | <0.001 |
| no | 82.0 | 78.0 | 68.3 |  |
| yes | 18.0 | 22.0 | 31.7 |  |
| N | 456 | 669 | 615 |  |
|  |  |  |  |  |
| **skin reflectance** |  |  |  | 0.31 |
| mean ± SD | 75.0 ± 2.8 | 75.0 ± 2.7 | 75.3 ± 2.7 |  |
| N | 183 | 313 | 367 |  |
|  |  |  |  |  |
| **skin colour score** |  |  |  | 0.18 |
| mean ± SD | 5.01 ± 1.24 | 5.05 ± 1.22 | 5.08 ± 1.21 |  |
| N | 1799 | 2490 | 2880 |  |
|  |  |  |  |  |
| **tanning score** |  |  |  | 0.20 |
| mean ± SD | 10.59 ± 2.15 | 10.63 ± 2.11 | 10.70 ± 2.10 |  |
| N | 1799 | 2490 | 2880 |  |
|  |  |  |  |  |
| **freckling score** |  |  |  | 0.27 |
| mean ± SD | 5.99 ± 1.71 | 6.03 ± 1.67 | 6.08 ± 1.72 |  |
| N | 1799 | 2490 | 2880 |  |
|  |  |  |  |  |
| **25(OH)D (nmol/l)** |  |  |  | 0.02/0.01^a^ |
| mean ± SD | 63.5 ± 21.1 | 63.8 ± 19.3 | 62.3 ± 18.9 |  |
| N | 1389 | 2209 | 2598 |  |

^a^P-value after adjustment for sex and age at blood draw.

Note: the O level was a secondary school leaving exam used in the United Kingdom from 1951 to 1988.

**Supplementary Table 5.** Pigmentation genetic scores and 25(OH)D levels according to sun avoidance behaviour in ALSPAC children.

|  | **mean ± SD of pigmentation score** | | |  | **mean ± SD** |  |
| --- | --- | --- | --- | --- | --- | --- |
|  | **skin color score** | **tanning score** | **freckling score** | **N^a^** | **25(OH)D (nmol/l)** | **N^b^** |
| **covering skin** |  |  |  |  |  |  |
| always | 5.10 ± 1.24 | 10.68 ± 2.15 | 6.11 ± 1.74 | 1222 | 62.6 ± 18.3 | 1117 |
| usually | 5.12 ± 1.21 | 10.71 ± 2.11 | 6.06 ± 1.72 | 2579 | 62.8 ± 18.9 | 2461 |
| sometimes | 4.91 ± 1.23 | 10.48 ± 2.06 | 5.98 ± 1.65 | 1670 | 63.7 ± 20.1 | 1606 |
| never | 4.94 ± 1.18 | 10.27 ± 1.83 | 5.63 ± 1.69 | 79 | 60.21 ± 22.49 | 74 |
| p-value | <0.0001 | 0.002 | 0.02 | 5550 | 0.20/0.11^c^ | 5258 |
|  |  |  |  |  |  |  |
| **wearing sun block** |  |  |  |  |  |  |
| always | 5.11 ± 1.24 | 10.70 ± 2.12 | 6.06 ± 1.70 | 3329 | 63.5 ± 19.3 | 3112 |
| usually | 5.00 ± 1.19 | 10.57 ± 2.08 | 6.05 ± 1.74 | 1765 | 62.4 ± 19.0 | 1698 |
| sometimes | 4.83 ± 1.15 | 10.37 ± 2.01 | 5.88 ± 1.56 | 452 | 61.5 ± 18.6 | 445 |
| never | 4.75 ± 1.11 | 10.01 ± 2.29 | 5.74 ± 1.86 | 43 | 61.6 ± 20.1 | 39 |
| p-value | <0.0001 | 0.001 | 0.12 | 5589 | 0.08/0.02^c^ | 5294 |
|  |  |  |  |  |  |  |
| **sun block factor** |  |  |  |  |  |  |
| 1-3 | 4.69 ± 1.02 | 10.32 ± 2.66 | 5.52 ± 1.38 | 6 | 56.0 ± 11.1 | 5 |
| 4-7 | 4.92 ± 1.08 | 10.05 ± 1.96 | 5.77 ± 1.76 | 70 | 66.2 ± 20.4 | 63 |
| 8-14 | 4.77 ± 1.22 | 10.47 ± 2.06 | 5.95 ± 1.76 | 374 | 63.0 ± 20.1 | 363 |
| 15-20 | 5.00 ± 1.20 | 10.53 ± 2.11 | 6.00 ± 1.66 | 1967 | 63.0 ± 18.7 | 1912 |
| 21-25 | 5.08 ± 1.23 | 10.72 ± 2.10 | 6.15 ± 1.74 | 964 | 63.3 ± 19.7 | 916 |
| >25 | 5.16 ± 1.23 | 10.75 ± 2.12 | 6.05 ± 1.72 | 2033 | 63.0 ± 19.2 | 1879 |
| p-value | <0.0001 | 0.001 | 0.14 | 5414 | 0.76/0.52^c^ | 5138 |

^a^N = number of individuals included in the analysis of pigmentation scores.

^b^N = number of individuals included in the analysis of 25(OH)D levels.

^c^Adjusted for sex and age at blood draw.

**Supplementary Table 6.** Association of pigmentation genetic scores with skin reflectance stratified by sex.

| **skin reflectance** | **effect^a^** | **95% CI** | **p-value** | **R^2b^** | **N** |
| --- | --- | --- | --- | --- | --- |
| **skin colour score** |  |  |  |  |  |
| males | 0.29 | 0.08, 0.51 | 0.01 | 0.019 | 370 |
| females | 0.52 | 0.28, 0.76 | 2.6x10^-5^ | 0.053 | 323 |
| p for interaction | 0.12 |  |  |  | 693 |
|  |  |  |  |  |  |
| **tanning score** |  |  |  |  |  |
| males | 0.16 | 0.04, 0.28 | 0.01 | 0.018 | 370 |
| females | 0.23 | 0.08, 0.37 | 0.002 | 0.029 | 323 |
| p for interaction | 0.52 |  |  |  | 693 |
|  |  |  |  |  |  |
| **freckling score** |  |  |  |  |  |
| males | 0.30 | 0.16, 0.45 | 7.4x10^-5^ | 0.042 | 370 |
| females | 0.16 | -0.02, 0.33 | 0.08 | 0.009 | 323 |
| p for interaction | 0.21 |  |  |  | 693 |

^a^Change in reflectance per unit increase in pigmentation score, adjusted for age at clinic visit and population stratification.

^b^Semipartial correlation coefficient squared, indicates the unique contribution of the genetic score to trait variability.

**Supplementary Table 7.** Association of pigmentation genetic scores with having freckles, stratified by sex.

| **any freckles** | **OR^a^** | **95% CI** | **p-value** | **R^2b^** | **N** |
| --- | --- | --- | --- | --- | --- |
| **skin colour score** |  |  |  |  |  |
| male | 1.63 | 1.33, 1.99 | 1.8x10^-6^ | 0.063 | 369 |
| female | 1.86 | 1.50, 2.31 | 2.2x10^-8^ | 0.097 | 344 |
| p for interaction | 0.22 |  |  |  | 713 |
|  |  |  |  |  |  |
| **tanning score** |  |  |  |  |  |
| male | 1.32 | 1.18, 1.48 | 1.7x10^-6^ | 0.063 | 369 |
| female | 1.44 | 1.27, 1.63 | 7.9x10^-9^ | 0.102 | 344 |
| p for interaction | 0.22 |  |  |  | 713 |
|  |  |  |  |  |  |
| **freckling score** |  |  |  |  |  |
| males | 1.40 | 1.22, 1.61 | 1.2x10^-6^ | 0.066 | 369 |
| females | 1.79 | 1.51, 2.12 | 1.1x10^-11^ | 0.152 | 344 |
| p for interaction | 0.02 |  |  |  | 713 |

^a^OR for having any freckles per unit increase in pigmentation score, adjusted for age at clinic visit and population stratification.

^b^Semipartial correlation coefficient squared, indicates the unique contribution of the genetic score to trait variability.

**Supplementary Table 8.** Time spent in the sun locally or abroad and 25(OH)D levels.

| **25(OH)D (nmol/l)** | **mean ± SD** | **N** |
| --- | --- | --- |
| **time in sun in UK at 5 years old** |  |  |
| no | 62.4 ± 18.6 | 995 |
| yes | 63.0 ± 19.3 | 3413 |
| p-value | 0.37/0.29^a^ | 4408 |
|  |  |  |
| **time in sun abroad at 5 years old** |  |  |
| no | 62.0 ± 18.8 | 2968 |
| yes | 65.0 ± 19.4 | 1407 |
| p-value | 9.1x10^-7^/2.1x10^-7a^ | 4375 |
|  |  |  |
| **time in sun in UK at 9 years old** |  |  |
| no | 62.3 ± 19.6 | 428 |
| yes | 62.4 ± 18.0 | 719 |
| p-value | 0.95/0.74^a^ | 1147 |
|  |  |  |
| **time in sun abroad at 9 years old** |  |  |
| no | 61.0 ± 18.0 | 941 |
| yes | 65.3 ± 20.2 | 330 |
| p-value | 0.0003/0.0001^a^ | 1271 |

^a^Adjusted for sex and age at blood draw.
